# Fibrosarcome naso-sinusien

**DOI:** 10.11604/pamj.2015.22.172.8124

**Published:** 2015-10-21

**Authors:** Madiha Mahfoudhi, Rim Lahiani

**Affiliations:** 1 Service de Médecine Interne A, Hôpital Charles Nicolle, Tunis, Tunisie; 2 Service d'ORL, Hôpital Charles Nicolle, Tunis, Tunisie

**Keywords:** Fibrosarcome, épistaxis, sinus maxillaire, Fibrosarcoma, epistaxis, maxillary sinus

## Image en medicine

Le fibrosarcome naso-sinusien est une tumeur maligne rare. Il se manifeste le plus souvent par une obstruction nasale et une épistaxis ou une cacosmie. Seuls les examens anatomo-pathologiques et immuno-histochimique permettent de confirmer le diagnostic. Le pronostic est péjoratif en cas de retard diagnostique. Patient âgé de 52 ans a consulté pour une obstruction nasale droite, une épistaxis récidivante et une altération de l’état général évoluant depuis deux mois et une douleur orbitaire. L'examen biologique a trouvé un discret syndrome inflammatoire et une anémie hypochrome microcytaire ferriprive avec hémoglobine à 10,5 g/dl. La TDM du massif facial a révélé une tumeur comblant le sinus maxillaire droit, d'aspect isodense avec rehaussement en cadre du sinus après injection du produit de contraste. Il a aussi objectivé une lyse de la paroi médiale du sinus maxillaire droit, du cornet moyen, du plancher orbitaire et de la lame papyracée avec extension du processus en extraconal. Plusieurs diagnostics ont été suspectés en particuliers un lymphome, une néoplasie ou une aspergillose. Il a bénéficié d'une exérèse totale de la tumeur à la fois par voie endoscopique et chirurgicale. Les examens anatomo-pathologiques et immuno-histochimique ont confirmé le diagnostic de fibrosarcome naso-sinusien. Le bilan d'extension était négatif. Le traitement a été complété par une radiothérapie adjuvante. L’évolution était marquée par l'absence de récidive ou de métastase avec un recul d'un an.

**Figure 1:**
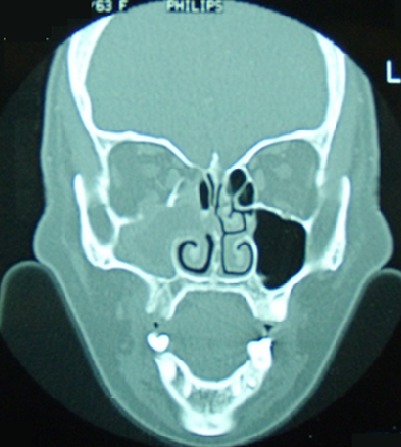
TDM du massif facial en coupe coronale: comblement d'aspect isodense du sinus maxillaire droit avec lyse de la paroi médiale du sinus maxillaire, du cornet moyen, du plancher orbitaire et de la lame papyracée et extension du processus en extraconal

